# Unraveling the immune response in optic nerve injury: implications for retinal ganglion cell protection

**DOI:** 10.3389/fimmu.2025.1671438

**Published:** 2025-12-01

**Authors:** Kexin Xu, Lu Yu, Ningzhi Zhang, Min Dong, Yiqiao Xing, Ning Yang

**Affiliations:** 1 Department of Ophthalmology, Renmin Hospital of Wuhan University, Wuhan, Hubei, China; 2 Department of Ophthalmology, The Second People’s Hospital of Jinzhou, Jinzhou, Hubei, China; 3 Department of Ophthalmology, Sir Run Run Shaw Hospital, Zhejiang University School of Medicine, Hangzhou, China; 4 Department of Ophthalmology, Aier Eye Hospital of Wuhan University, Wuhan, Hubei, China

**Keywords:** immunity, optic nerve, retinal ganglion cell, blood-retinal barrier, T cell receptor signaling

## Abstract

Optic nerve injury (ONI) initiates complex immune responses that can act as a “double-edged sword,” promoting either neuroprotection or neurodegeneration of retinal ganglion cells (RGCs). In this review, we integrate evidence on both innate and adaptive immunity in ONI, emphasizing the dual roles of microglia, Müller cells, astrocytes, T and B lymphocytes, and the complement system. While glial activation and blood–retina barrier breakdown are critical determinants of local inflammation, T-cell response, which are shaped by subset composition, antigen specificity, and checkpoint signaling, can further shift the balance between repair and injury. Recent advances, including single-cell and spatial transcriptomic analyses, as well as experimental modulation of immune checkpoints, reveal new opportunities—such as precise immune mapping, checkpoint-targeted neuroprotection, and gene-based immunoregulation—but also persistent challenges, including the need to clarify the spatiotemporal dynamics of immune activity, overcome interspecies differences between rodent and human models, and ensure the safety of immunomodulatory strategies in the immune-privileged eye. By applying the “double-edged sword framework” consistently across these immune mechanisms, we highlight how cellular context and timing determine divergent outcomes. Finally, we discuss emerging approaches such as regulatory T-cell enhancement, targeted inhibition of complement overactivation, senolytics, and gene-editing interventions, outlining translational perspectives for immune-guided neuroprotection in ONI.

## 1 Introduction

Optic nerve injury (ONI) is a major cause of irreversible vision loss, primarily due to the degeneration of retinal ganglion cells (RGCs) (1). The pathophysiology of ONI involves not only direct axonal damage but also complex secondary processes driven by immune and glial responses (2).

Recent evidence has increasingly highlighted that immune responses function as a double-edged sword: while acute activation of protective mechanisms may facilitate debris clearance, axonal regeneration, and neurotrophic support, chronic or dysregulated activity often results in neuroinflammation, blood–retina barrier (BRB) disruption, and accelerated RGC death. Framing ONI immunity within this dualistic paradigm provides a unifying perspective to reconcile findings that otherwise appear contradictory (3, 4).

In particular, the seemingly contradictory roles of T lymphocytes (T cells) exemplify this duality. Their dual functions should not be interpreted as a simple dichotomy, but rather as the result of functional plasticity shaped by species differences, injury models (e.g., acute ischemia and chronic glaucoma), temporal dynamics of immune infiltration, and subset composition (e.g., T helper 17 [Th17] vs. regulatory T cells [Treg]). Furthermore, anatomical localization (retina versus optic nerve head) and antigen-specific interactions, including immune checkpoint regulation on neurons such as programmed cell death 1 (PD-1) expression on RGCs, critically determine whether T-cell activity becomes neuroprotective or pathogenic.

Microglia, as resident innate immune cells, rapidly respond to axonal injury. They can phagocytose debris and secrete growth factors that transiently support neuronal survival. However, prolonged activation leads to the release of pro-inflammatory cytokines such as tumor necrosis factor (TNF)-α and interleukin (IL)-1β, which exacerbate neuronal degeneration. Müller cells, another key retinal glial population, contribute to maintaining BRB integrity and metabolic support under physiological conditions. However, under stress, they amplify inflammatory cascades and promote gliosis. Astrocytes similarly show dual functions, providing structural and metabolic support early after injury but later contributing to scar formation and neurotoxicity (5–9).

Adaptive immune cells also display this duality. Tregs have been shown to promote neuroprotection through IL-10 and transforming growth factor (TGF)-β secretion, whereas Th17 and CD8^+^ T cells may drive persistent inflammation and RGC apoptosis in glaucoma and optic nerve crush models. Immune checkpoint pathways, including PD-1 and cytotoxic T-lymphocyte-associated protein 4 (CTLA-4), further modulate these responses, raising both therapeutic opportunities and safety concerns (10–13).

Together, these findings underscore that the impact of immune activity on ONI depends on cellular identity, anatomical localization, temporal dynamics, and context-dependent signaling pathways. In this review, we aimed at determining the innate and adaptive roles of immune cells in ONI by systematically analyzing how these cells contribute to RGC loss or survival. We focus on key cellular interactions between microglia and Müller cells, and explore how the breakdown of ocular immune privilege influences disease progression. We also discuss emerging therapeutic strategies that could manipulate immune pathways for neuroprotection and regeneration. We provide a comprehensive explanation of current concepts and identify new directions for investigation and clinical intervention in ONI.

## 2 Immune cells

### 2.1 Innate immunity

Eyes are highly intricate and vital organs that play crucial roles in facilitating human interaction with the environment. Despite continuous exposure to harmful stimuli, such as infectious agents, pollutants, and mechanical stress, the eyes have evolved robust defense mechanisms. This includes innate immunity, which serves as the first line of defense against pathogens.

Innate immunity is characterized by an immediate physiological response to pathogens or foreign objects, typically occurring within minutes to 96 h after exposure. At the ocular surface, innate defenses such as antimicrobial peptides, lysozymes, and lactoferrin in tear fluid protect against external pathogens. By contrast, in the posterior segment, and specifically within the retina, innate immune activity is organized around glial networks (retinal microglia, Müller cells, and astrocytes), which differ fundamentally from ocular-surface innate defenses in composition, localization, and immune privilege constraints (BRB) (14).

Innate immunity is distinguished by a broad range of actions, rapid responses, relative stability, and heritability that collectively establish the foundation for specific immunity.

#### 2.1.1 Innate immune cells in retinal and optic nerve protection

Innate immune cells are the first line of defense in the retina and optic nerve, both providing acute protection and being potential drivers of chronic neuroinflammation. They include phagocytes (neutrophils and mononuclear phagocytes), dendritic cells (characterized by extensive branching processes and widespread distribution throughout tissues and organs), natural killer T cells (originating from bone marrow lymphoid stem cells), gamma delta T cells, B-1 cells, and mast cells, as well as eosinophils and basophils. They play roles in clearing damaged, senescent, and aberrant cells and contribute to adaptive immune responses.

In the retina, the predominant cytokine producers are glial cells, particularly microglia, Müller cells, and astrocytes. Microglia and macrophages are pivotal innate immune cells located in the retina and choroid, where they phagocytose apoptotic cells and debris to maintain homeostasis (3, 15, 16). Retinal microglia, distributed in the ganglion cell layer and plexiform layers, are highly responsive to injury and release inflammatory cytokines such as IL-1β and TNF-α (14, 17, 18). Müller cells provide structural and metabolic support and also release ATP under stress, thereby perpetuating microglial activation (19–22).

RGCs themselves are not considered primary immune effectors. Under physiological conditions, they contribute minimally, but under stress or disease, they can upregulate pattern-recognition receptors such as toll-like receptors (TLRs) and secrete cytokines in a context-dependent manner (23–27). However, compared with glia, their contribution to cytokine production is limited.

Microglial activation remains a key indicator of neurodegenerative severity, with reduced activation correlating with preserved optic nerve integrity. In ONI, activated microglia selectively target damaged neurons and synapses for phagocytosis and complement-mediated clearance. While these processes are essential for early debris removal, excessive or chronic activation contributes to synaptic degeneration, axonal disruption, and irreversible vision loss (5–9, 28, 29).

Recent single-cell RNA sequencing studies have revealed profound heterogeneity among retinal glia, identifying subsets of microglia and Müller cells with divergent inflammatory or neurotrophic profiles (30, 31). This heterogeneity helps explain why findings from bulk studies sometimes appear contradictory. Translating these insights into therapy is challenging: targeting glial subtypes requires precise spatiotemporal modulation, and cross-species differences between rodent and human retina complicate direct extrapolation.

In summary, innate immune cell activity in ONI is characterized by a dynamic balance: glial-derived cytokines and phagocytic activity are essential for acute protection, whereas chronic activation of the same pathways promotes RGC loss. RGCs themselves can contribute under stress conditions; however, their role is secondary to that of glia.

#### 2.1.2 Innate immune molecules

The complement system is an essential component of the innate immune response (32) that consists of more than 30 distinct enzymatically active proteins in the serum and tissue fluids in humans and other vertebrates (33). Although primarily synthesized by hepatocytes, mononuclear macrophages, endothelial and intestinal epithelial cells, and keratinocytes also produce complement proteins (34, 35). Complement activation is initiated by four principal pathways. The classical pathway mediated by antigen-antibody complexes leads to the activation of complement components (C) 1, 4, 2, and 3, which results in the formation of C3 and C5 convertases that cleave C5. This ultimately causes the formation of C5b-9 membrane attack complexes (MACs) that create pores in cell membranes, resulting in osmotic imbalance and cell lysis. The Mannan-binding lectin pathway is activated during the early stages of pathogen infection. Mannan-binding lectin binds to bacterial mannose residues that activate a serine protease complex with activity similar to that of C1q, initiating a cascade reaction analogous to the classical pathway. The alternative pathway is activated by bacteria, bacterial endotoxins, glucans, yeast polysaccharides, and agglutinated immunoglobin (Ig)A and IgG4. The complement system operates as an interactive network of proteins that rapidly neutralizes microbial invaders or endogenous stress signals through opsonophagocytosis and inflammation (36). It contributes to tissue homeostasis and host immune surveillance by coordinating innate and adaptive immune signals. Both C3 and C5 are pivotal components of the complement cascade. C3 has emerged as a significant therapeutic target in inflammatory diseases such as age-related macular degeneration (37, 38). It is involved in cell regulation and plays a vital role in recruiting microglia and macrophages and in modulating downstream pathways (39).

Although the complement system facilitates pathogen clearance and debris removal, its overactivation in the retina might induce neuronal injury. When C3 is upregulated, downstream MACs form in ONI, as found in experimental animal models and human glaucomatous tissues (40). While C3b-mediated opsonization helps to clear apoptotic RGCs, uncontrolled MAC formation disrupts the integrity of the neuronal membrane, which directly contributes to RGC death. This duality highlights the delicate balance between complement-mediated protection and neurotoxicity in ONI pathogenesis.

Therapeutics targeting complement components are promising treatment modalities in that C3 inhibitors, such as pegcetacoplan, can reduce retinal inflammation and lesion progression in geographic atrophy secondary to age-related macular degeneration (41). Although glaucoma-specific trials are still ongoing, preclinical findings of C3-targeted gene therapy have reduced axonal and neuronal degeneration in models of chronic glaucoma (42). Modulating complement regulators or enhancing endogenous inhibitors (such as factor H) might preserve retinal function in ONI, minimizing neurotoxic effects while maintaining essential immune surveillance (40).

In conclusion, ocular innate immunity plays critical roles, particularly innate immune cells and RGCs. These findings enhance our understanding of ocular diseases and might facilitate the development of innovative therapeutic strategies. Future studies should explore interactions between the ocular and corporeal immune systems to potentially contribute to the treatment of other systemic conditions.

### 2.2 Adaptive immunity

Adaptive immunity is activated in response to sustained pathogen exposure. Although the establishment of adaptive immunity typically requires 1 to 2 weeks, it is characterized by the specific recognition of pathogens and the generation of immunological memory, rendering it essential for defense against external threats (43). T cells are integral to adaptive immunity (44, 45).

#### 2.2.1 T cells

T cells are integral to adaptive immunity and are classified into naïve, regulatory, effector, and memory subsets, which together provide protection and maintain immune homeostasis throughout life (46). Their activation and differentiation are tightly regulated by signaling and metabolic cues, including mechanistic target of rapamycin (mTOR), glucose and glutamine metabolism, and co-stimulatory pathways such as CD28, which govern the transition from quiescence to functional specialization (47–50). While these fundamental mechanisms are well established, their implications in the retina warrant closer examination.

In the context of ONI, the relevance of T cells lies in how distinct subsets interact with retinal cells to shape outcomes. Microglia, as the first line of defense, recognize damage-associated signals and present antigens to T cells (14). Depending on subset composition, T cells can directly release cytotoxic factors (e.g., interferon [IFN]-γ, IL-17) that exacerbate RGC injury or exert immunoregulatory effects through IL-10 and TGF-β to restrain inflammation and maintain tolerance. Importantly, RGCs themselves may also modulate immune activity: in chronic glaucoma models, aberrant expression of PD-1 on RGCs promoted the transformation of microglia toward an M2 phenotype, thereby conferring neuroprotection (i.e., protective effects of blocking PD-1 pathway on RGCs in a mouse model of chronic ocular hypertension).

These findings indicate that the role of T cells in ONI cannot be understood in isolation but must be interpreted through the lens of subset identity, microglial antigen presentation, and neuronal checkpoint regulation. Further exploration of these interactions may be key to revealing mechanisms of optic nerve diseases and developing new therapeutic strategies.

#### 2.2.2 T-cell receptor

T cell activation is initiated by the specific binding of major histocompatibility complexes (MHC) to T-cell receptor (TCR)αβ heterodimers that are stabilized by CD8 and CD4 binding to MHC classes I and II, respectively (51). TCRαβ heterodimers are associated with multiple signal transduction subunits (CD3γ, CD3δ, CD3ϵ, and CD3ζ) that activate downstream signaling pathways. These subunits contain tyrosine-based immune receptor activation motifs that recruit proteins with SH2 domains, such as tyrosine protein kinase zeta-chain-associated protein kinase 70 (ZAP70) (52). The affinity, duration, and intensity of TCR signaling determines the metabolic and functional programs of T cells. The role of TCRs in ONI and repair remains equivocal. TCRs are pivotal for antigen recognition and signal transduction, initiating immune responses (53). A critical component of TCRs is CD3ζ, which is expressed by mouse RGCs and amacrine cells to regulate their development. The loss of CD3ζ impairs RGC axon projections into the dorsal lateral geniculate nucleus and normal amacrine cell development in the retina (54, 55). Molecules related to TCR can modulate immune cell morphology and function. For example, CD3ζ activation influences the cytoskeleton, thus regulating immune cell morphology. Similar to neurons, immune molecules might function as signals that affect synaptic growth, development, morphology, and activity (55), and might play novel roles in neuronal function and development.

**Figure 1:**
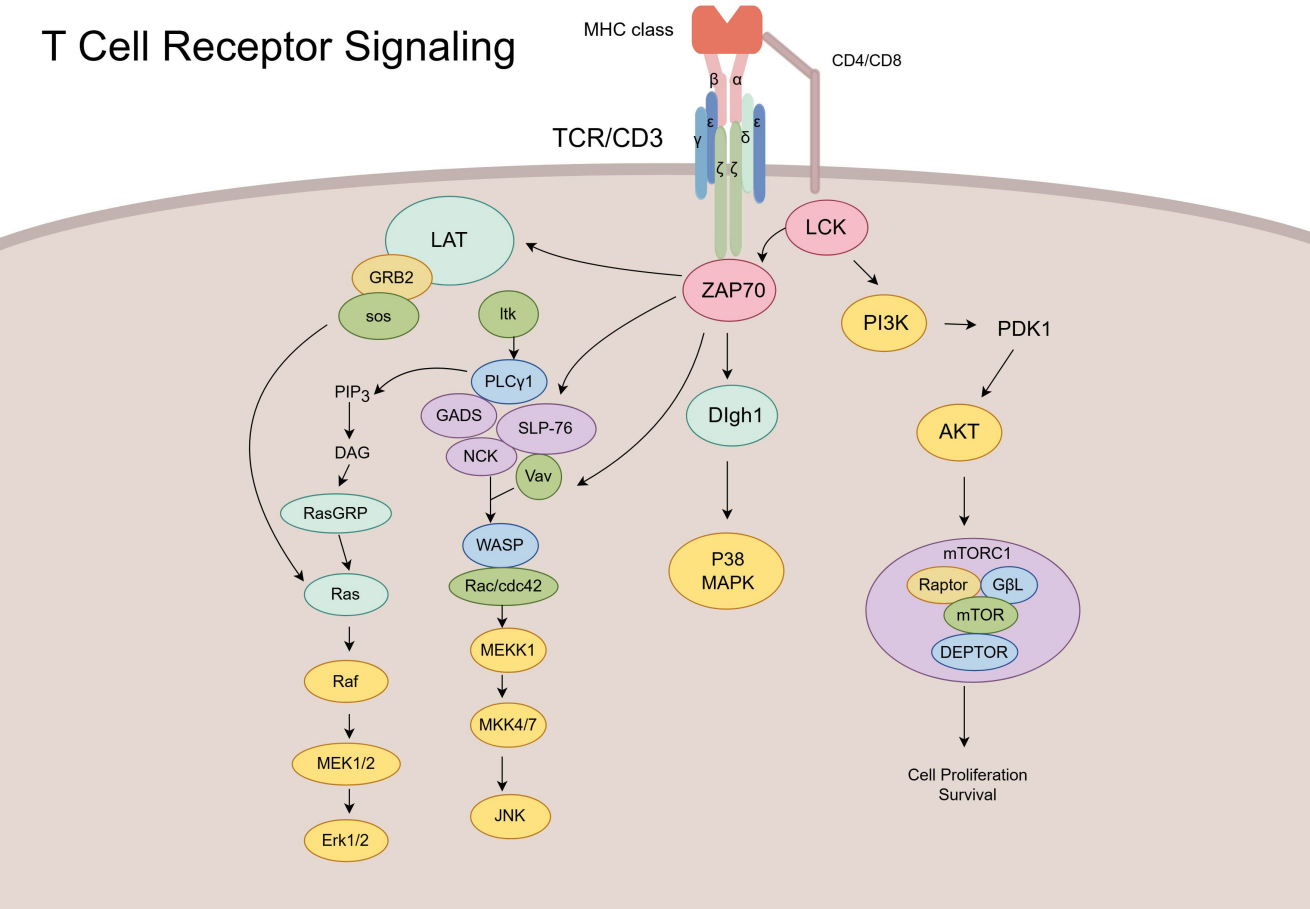
Schematic of T-cell receptor (TCR) signaling pathways and downstream effector cascades. Upon antigen recognition, the TCR/CD3 complex activates lymphocyte-specific protein tyrosine kinase (LCK) and ZAP70, initiating multiple downstream signaling cascades. These include the LAT–GRB2–Ras–ERK pathway, PLCγ1-mediated pathway, PI3K–AKT–mTOR axis, and Vav–Rac–JNK module. Collectively, these pathways regulate T-cell activation, proliferation, cytoskeletal reorganization, and effector functions.

By recruiting various adaptor and skeletal proteins, TCR activation affects pathways such as the main branches of the mitogen-activated protein kinase (MAPK) signaling pathway, including the P38, extracellular signal-regulated kinase (ERK), Jun N-terminal kinase (JNK), and phosphoinositide 3 kinase (PI3K)-AKT-mTOR signaling pathways, which regulates cell survival, proliferation, and differentiation (56, 57). Activation of the p38/MAPK pathway by ZAP70 requires the participation of discs large homolog 1, which is an alternative approach (58). Unlike the dual phosphorylation of P38 in the classical activation pathway, P38 is phosphorylated *via* the monophosphorylation of TCRs, which might affect T-cell function. ZAP70 recruits linker for activation of T cells to promote rat sarcoma (RAS) and guanylate releasing protein, which influences activation of the Erk/MAPK pathway (**Figure 1**) (59). The TCR signaling pathway is strictly regulated by various proteins, and different functions of T cells are regulated throughout the life process. Therefore, understanding the influence of immune molecules in TCR is important for understanding the function of T cells and treating various diseases.

#### 2.2.3 T-cell co-signaling molecules

The fate of naïve T cells is determined not only by TCR-MHC interactions but also by co-signaling molecules on their surface. These molecules, which include both co-stimulatory and co-inhibitory factors, are collectively referred to as immune checkpoints (72, 73). Most T-cell co-signaling molecules belong to the Ig or tumor necrosis factor receptor superfamilies. Co-stimulatory factors are essential for T-cell activation. In their absence, even if the TCR is activated, T cells cannot proceed with critical processes such as signal transduction, subpopulation differentiation, and the production of inflammatory factors (72, 74). The most pivotal co-stimulator is CD28, which belongs to the Ig superfamily. It reduces the T-cell activation threshold and facilitates the recruitment of molecules such as PI3K and lymphocyte-specific protein tyrosine kinase (75), thereby driving transcriptional programs and effector function (74).

Co-inhibitory molecules are crucial for maintaining cellular homeostasis and preventing excessive tissue damage. Notable examples include T-cell Igs, mucin domain-containing 3, cytotoxic T-lymphocyte antigen 4, and PD-1 (10, 11). CTLA-4 competes with CD28 to bind CD80 and CD86 ligands with greater affinity. It inhibits the cell cycle by suppressing the AKT signaling pathway and degrading tryptophan (12, 13). These checkpoints are minimally expressed on naïve T cells but rapidly upregulated upon activation, providing a feedback mechanism to prevent overactivation (76).

In the setting of ONI, the balance between co-stimulatory and co-inhibitory signaling is a decisive factor in determining RGC fate. Excessive CD28-mediated activation enhances pro-inflammatory T-cell responses and microglial reactivity, which aggravates RGC loss in rodent glaucoma models. By contrast, PD-1 and CTLA-4 signaling can restrain detrimental T-cell activity and protect RGCs from secondary damage (77). Notably, PD-1^+^ T cells have been detected in the human glaucomatous retina, underscoring the translational relevance of checkpoint pathways (30, 78). However, systemic checkpoint blockade, while beneficial in cancer, may exacerbate retinal inflammation if applied indiscriminately. Thus, T-cell co-signaling molecules exemplify the “double-edged sword” nature of immunity in ONI, offering potential neuroprotection when appropriately modulated, but contributing to degeneration when dysregulated.

#### 2.2.4 T cells and ONI

Infiltration of T cells into the retina exerts diverse effects on RGCs and optic nerves. Retinal T cells might exacerbate optic nerve damage and contribute to vision loss. Acute injury induced T-cell infiltration into the retina that promoted the persistent loss of RGCs in a mouse model of glaucoma (79). The pathogenesis of glaucoma might be associated with heat shock proteins (HSP), as HSP27 and HSP60 expression is increased in clinical samples of patients with glaucoma (31) (80). HSP60-induced reduction in RGCs and axons might occur through Fas/FasL pathway signaling, which has been shown mainly in *in vivo* mouse experiments (62). Chen et al. suggested that the long-term loss of RGCs and axons after glaucoma in mice is associated with HSP-related T cells, specifically IFN-γ-secreting CD4+ T cells that infiltrate and damage RGCs in murine experimental glaucoma models (63). Neurodegeneration in glaucoma might result from interactions between T cells and microglia that they activate, leading to the release of substances harmful to RGCs, such as TNF-α and nitric oxide synthase-2, as validated in both rodent models and human post-mortem glaucoma tissues (64). Conversely, T cells might play a neuroprotective role in ONI, because CD4^+^CD25^+^ T cells upregulate and increase RGC survival in optic nerve transection model rats in *in vivo* studies (3, 30). The transfer of activated T cells to rats following optic nerve compression demonstrated neuroprotective effects against secondary degeneration (81). T cells play dual roles in the central nervous system of experimental animal models (82). Their potential for nerve repair and adverse effects emphasizes their clinical therapeutic value.

Importantly, the apparent contradiction between neuroprotective and neurotoxic roles reflects not only T-cell subset heterogeneity (e.g., Tregs vs. Th17 vs. CD8^+^ cells) but also the timing and anatomical context of immune infiltration (60, 83). Protective effects of Tregs have been consistently observed in acute injury paradigms, while pathogenic CD4^+^ and CD8^+^ subsets dominate in chronic glaucoma models. However, direct validation in human tissue remains limited and often relies on postmortem analysis, which cannot capture dynamic immune kinetics.

Future investigations should prioritize dissecting the spatiotemporal dynamics of T-cell responses, employing longitudinal *in vivo* imaging and single-cell profiling to map subtype-specific effects (84, 85). Moreover, the translational potential of immune checkpoint modulation (e.g., PD-1, CTLA-4) must be carefully evaluated for ocular contexts, balancing neuroprotection against systemic immunosuppression risks. Addressing these knowledge gaps will be essential to determine whether T-cell-targeted interventions can be harnessed as viable therapies for ONI **Table 1**.

**Table 1:** Protective vs. detrimental immune pathways in optic nerve injury.

Immune component	Protective effects	Detrimental effects	Type of evidence
T cells	Tregs promote RGC survival via IL-10/TGF-β; constrain microglia activation (3, 30, 60, 61)	Th17/CD8^+^ amplify inflammation and cytotoxicity; HSP-reactive T cells drive chronic RGC/axon loss (62–66)	*In vivo* mouse ONC/glaucoma; Human HSP expression/serology
Microglia	Acute debris clearance; transient neurotrophic support (19–22)	Chronic activation → TNF-α/iNOS, BRB breakdown, synapse loss (5, 6, 9, 29, 37)	*In vivo* rodent ONI/glaucoma; Human post-mortem summary
Müller cells	BRB maintenance; neurotrophic factor production (67–69)	Prolonged reactivity → cytokine surge, gliosis (29, 70, 71)	*In vitro* primary/cell line; *in vivo* rodent glaucoma; scRNA-seq Human
Astrocytes	Structural/repair support (17, 69)	Reactive astrocytes via IL-1α/C1q/TNF-α promote neurotoxic microglia (29, 31, 71)	*In vivo* ONC/glaucoma; human DR/GL postmortem
Complement system	C1q-mediated developmental synaptic pruning	C3 overactivation → chronic neuroinflammation/RGC loss	Mouse glaucoma/degeneration; human glaucoma tissue

**Figure 2:**
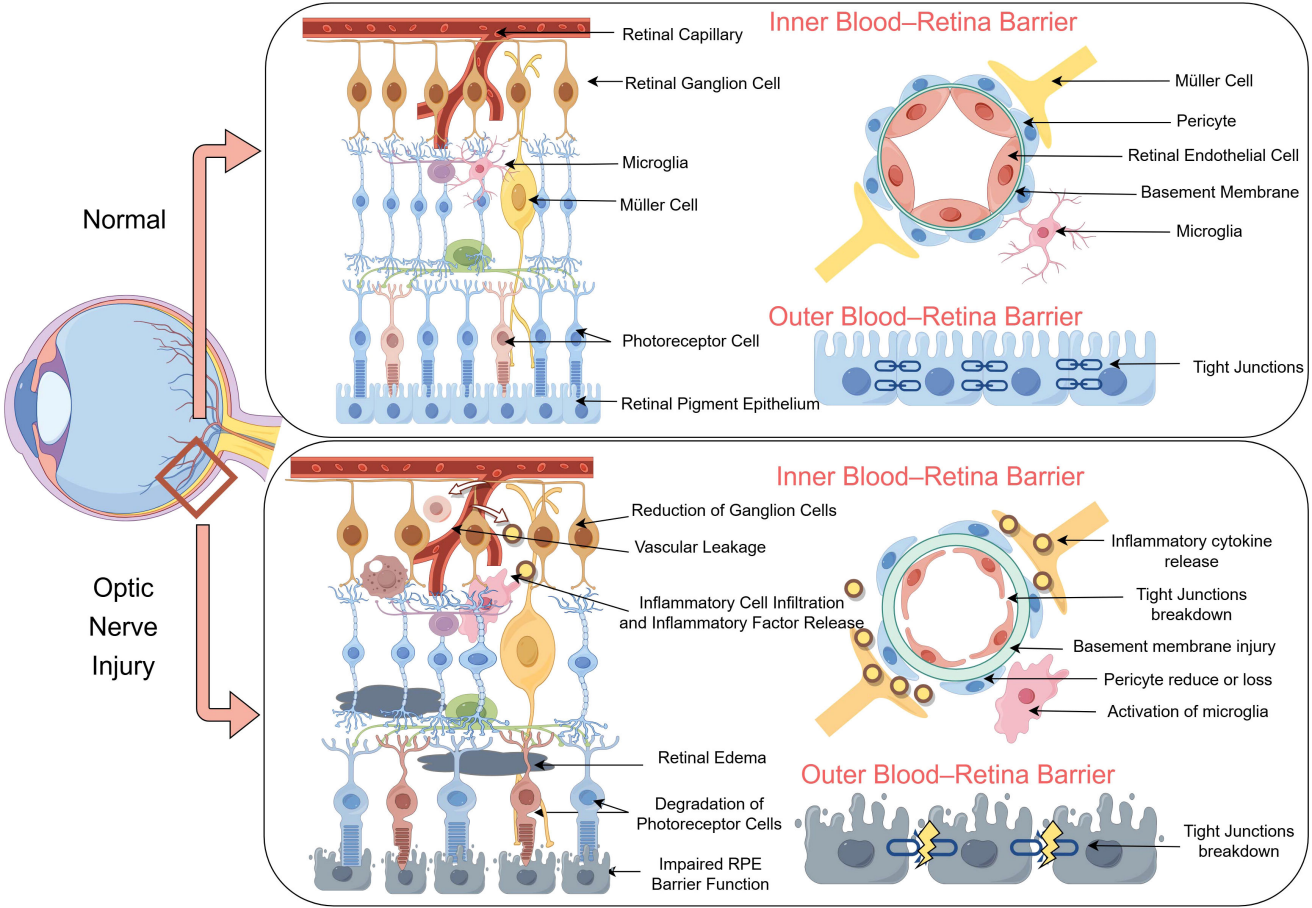
Structural and functional disruption of the blood–retina barrier (BRB) following optic nerve injury (ONI). The schematic illustrates a comparison between normal (top) and ONI (bottom) conditions of the retina, highlighting alterations in the inner and outer BRB. Under normal conditions, retinal capillaries are ensheathed by intact endothelial cells, pericytes, and Müller cells, with preserved tight junctions and organized neurovascular units. In contrast, ganglion cell loss, microglial activation, and inflammatory cell infiltration following ONI lead to cytokine release, tight junction breakdown, and basement membrane injury. Vascular leakage and pericyte loss further compromise the integrity of the BRB. In the outer BRB, photoreceptor cell degeneration and impaired retinal pigment epithelium barrier function are accompanied by disorganized tight junctions, which contribute to retinal edema and neuroinflammation.

Dual (double-edged) roles of major immune components involved in optic nerve injury, including T cells, microglia, Müller cells, astrocytes, and the complement system. For each component, both protective and detrimental effects are listed alongside the predominant type of supporting evidence (*in vitro*, *in vivo*, or human postmortem/clinical studies). References correspond to those cited in the main text. The table complements **Figure 2** by providing a structured comparative overview, allowing evaluation of context-dependent immune functions and the translational relevance of different findings.

#### 2.2.5 B cells

B cells contribute to adaptive immunity through antigen presentation, antibody secretion, and the release of anti- or pro-inflammatory factors (86). Similar to T cells, B cells rarely enter the normal central nervous system (CNS), but can cross the barrier in pathological states (86, 87). Although little is known about B cells in ONI, they serve as therapeutic targets in CNS diseases such as multiple sclerosis. The results of therapies targeting CD20^+^ B cells have been significant in patients with multiple sclerosis (87). Interactions between various immune cells, including T and B cells, affect their secretion and activation (87). Given that the optic nerve is a part of the CNS, investigating B cells might provide new insights into ONI treatment strategies.

### 2.3 Ocular and retinal immune privileges

The eyes have distinct immune privileges that are facilitated by various mechanisms. The BRB is integral to maintaining retinal homeostasis and function (88). The retina suppresses immune cell activation and promotes immunosuppression *via* diverse neuro-modulatory proteins (89). Ocular immunity encompasses the anterior chamber, vitreous cavity, and posterior chamber, thereby inducing systemic immune bias (70). It not only induces systemic immunosuppression and reduces the occurrence of immune responses but also facilitates eye treatment (71). The BRB comprises inner and outer components that isolate the retina from the systemic circulation, thus preventing inflammatory cell migration and ensuring retinal stability (67). The inner BRB consists of capillary endothelial cells, pericytes, and Müller cell protrusions that modulate retinal endothelial cell activity (68, 69). The outer BRB is characterized by tight junctions between adjacent pigment epithelial cells, and it regulates capillary transport and blood solutes (88, 90).

Neurovascular units (NVUs) constitute the structural foundation of the BRB and regulate blood component access to the retina. The retinal NVU comprises the vascular system (endothelial cells and pericytes), macroglia (astrocytes and Müller cells), neurons (ganglion, anoplectic, and horizontal cells), and immune cells (microglia and macrophages). Astrocytes and microglia form surface NVUs in the superficial capillary plexus, whereas neurites without long cells and glia, such as microglia, form intermediate nerve plexus NVUs. Microglia envelop horizontal cells to form deep nerve plexus NVUs.

A disrupted BRB disrupts retinal homeostasis due to immune cell infiltration and inflammatory mediators (91). RGCs produce vascular endothelial growth factor, which modulates the BRB (88). Under physiological conditions, immune cells do not penetrate the BRB or reach the retina. However, immune cell extravasation into the retina is associated with various ocular diseases, including glaucoma, uveitis, and diabetic retinopathy (65, 92, 93). In instances of BRB damage, such as elevated intraocular pressure, T cells can infiltrate the retina (30, 63, 66). The BRB and T cells play pivotal roles in the pathogenesis of retinal and optic nerve diseases. Evaluating T-cell responses to retinal injury might reveal novel therapeutic targets for the treatment of retinal degeneration (**Figure 2**). The mechanisms underlying ocular immune privilege are progressively compromised in the context of ONI. A disrupted BRB facilitates the infiltration of peripheral immune cells and complement factors into the retinal parenchyma, creating a pro-inflammatory environment. Müller cell dysfunction and pericyte loss destabilize the NVU, further impairing the immunosuppressive microenvironment. Upregulated MHC molecules and lost checkpoint regulation might trigger autoreactive responses that sustain chronic inflammation and accelerate RGC loss.

Various immune cells can inflict irreversible damage on the retina and optic nerves by compromising the ocular immunosuppressive barrier. Investigating the mechanisms underlying this destructive process might lead to new strategies for protecting the optic nerve and mitigating disease severity. Furthermore, the collapse of immune privilege in ONI complicates therapeutic strategies that rely on retinal immune quiescence (94). Cell or gene therapy might be less effective in inflamed retinal environments. Future approaches might need to include adjunctive strategies to restore BRB integrity and re-establish immune tolerance, such as modulating IL-10, TGF-β, or vascular endothelial growth factor signaling. Preserving or reconstructing ocular immune privileges might enhance both neuroprotection and therapeutic responsiveness in ONI (95).

## 3 Discussion

Current clinical interventions for ONI, including glaucoma, remain suboptimal and highlight the urgent need for more effective therapies. Increasing evidence shows that immune responses act as a double-edged sword: while timely activation of glia and T cells can clear debris, promote axonal repair, and preserve BRB function, prolonged or dysregulated activation accelerates RGC degeneration. The integration of advancements in immune cell research with cell, anti-aging, and gene therapies might present innovative strategies for mitigating RGC loss and improving visual outcomes (96, 97).

Aging, immune cells, and RGCs are intricately interconnected (97). Aging increases vulnerability to diseases and diminishes the effectiveness of immunotherapy. Immunosenescence, characterized by elevated pro-inflammatory levels, metabolic dysregulation, and reduced T-cell input, is associated with various ocular diseases, including glaucoma and diabetic retinopathy (98). Most of these findings have been obtained based on *in vivo* mouse models of experimental glaucoma or ocular hypertension, whereas validation in human aging cohorts and postmortem retinal tissues remains sparse. Immune cells undergo diverse changes with aging, such as the loss of TCR diversity, microglial transformation into pro-inflammatory cells, and RGC destruction (99, 100).

While elevated intraocular pressure (IOP) significantly contributes to glaucoma development, aging is also a critical factor (101). Rates of RGC survival are lower in older than in younger mice at equivalent IOP levels (99, 101). Elevated IOP leads to the generation of senescence-associated phenotypes in RGCs, and promotes apoptosis and senescence in peripheral RGCs. The removal of senescent cells can effectively protect RGCs and maintain visual function (99).

Substantial translational challenges remain. Most senolytic and cryopreservation approaches are confined to *in vitro* and preclinical settings. Cell cryopreservation and anti-aging approaches can potentially treat senescent immune cells and RGCs. Current cryopreservation technology has advanced as stem cell-derived or cryopreserved RGCs have been transplanted into the retina (102, 103). However, genetic changes and immune cell cytotoxicity during cryopreservation are obstacles to the development of cell-specific immunotherapy, and connections between original and transplanted neurons need to be strengthened (103, 104). These observations are currently limited to *in vitro* systems and preclinical animal models, with no human clinical applications reported to date. Cryopreserved immune cells might help preserve immunological function in ONI. The reintroduction of cryopreserved immune cells with enhanced neuroprotective phenotypes could also counteract immune dysregulation and facilitate retinal repair.

Senolytic therapies that selectively target senescent cells might protect RGCs and maintain visual function. Anti-aging drugs, such as dasatinib, can reduce DNA damage, reactive oxygen species, and inflammatory cytokines, thereby protecting the retina and reducing RGC death without significant adverse effects (99, 105). Nevertheless, the systemic safety profile of these drugs and ocular-specific delivery strategies require careful evaluation before translation to clinical settings. Cellular senescence is widespread in multiple corporeal systems, and anti-aging drugs can target a variety of diseases and provide considerable health benefits.

Advancements in cellular, genetic, and protein analyses have led to the identification of neuroprotective immune cell subsets and their beneficial genes (106, 107). Gene-editing technologies, such as CRISPR/Cas9 and base-editing systems, enable precise modulation of immune pathways to enhance tissue regeneration and neuroprotection. To date, these strategies have remained restricted to animal models, without human clinical translation, underscoring a critical research bottleneck.

For example, downregulated pro-inflammatory effectors such as TNF-α or C3 in microglia, or enhancement of CD4^+^CD25^+^ regulatory T cells, might reduce neuroinflammation and preserve BRB function. Inhibiting TNF-α protects the BRB by preventing cytokine-induced vascular permeability (108). Likewise, the genetic deletion of complement C3 in microglia reduced neuroinflammatory damage in retinal degeneration models (109). Moreover, CD4^+^CD25^+^ regulatory T cells exert neuroprotective effects by secreting anti-inflammatory cytokines and mitigating RGC loss in ONI models (61). The expression of CD3ζ in RGCs similarly supports dendritic and axonal connectivity, improving neuronal survival and synaptic refinement (55).

In summary, the central challenge in ONI is to balance protective and detrimental immunity. Future investigations should focus on tailoring immune-based gene therapies to optimize optic nerve repair. By leveraging immune regulation and regenerative medicine, novel interventions can achieve functional optic nerve restoration and visual rehabilitation.

## 4 Conclusion

Immune responses in ONI embody a double-edged sword: under appropriate conditions, microglia, Müller cells, T cells, and astrocytes contribute to tissue repair, neuroprotection, and BRB preservation, whereas chronic activation or loss of regulation accelerates degeneration through cytokine release, complement overactivation, and checkpoint dysregulation. Our review highlights the dual nature of these immune pathways, integrating evidence from innate and adaptive immunity, aging and immunosenescence, and therapeutic modulation.

Recent progress has revealed protective mechanisms such as the neurotrophic effects of Müller cells, the regulatory capacity of Tregs, and the BRB-stabilizing function of CD3ζ. Conversely, detrimental mechanisms include chronic microglial activation, Th17- and CD8^+^-mediated cytotoxicity, complement-driven neuroinflammation, and astrocyte-derived neurotoxicity. Advances in single-cell transcriptomics have further emphasized the heterogeneity of retinal immune cells, underscoring the importance of the spatiotemporal context in shaping outcomes.

Future research has to define the spatiotemporal rules of immune activity and develop targeted interventions that amplify protective immunity while restraining harmful responses. By reframing ocular immunity as a double-edged sword, the field can shift from descriptive studies toward rational, mechanism-based therapies for optic nerve protection and regeneration.
